# BCMA- and CST6-specific CAR T cells lyse multiple myeloma cells and suppress murine osteolytic lesions

**DOI:** 10.1172/JCI171396

**Published:** 2024-01-02

**Authors:** Fumou Sun, Yan Cheng, Jin-Ran Chen, Visanu Wanchai, David E. Mery, Hongwei Xu, Dongzheng Gai, Samer Al Hadidi, Carolina Schinke, Sharmilan Thanendrarajan, Maurizio Zangari, Frits van Rhee, Guido Tricot, John D. Shaughnessy, Fenghuang Zhan

**Affiliations:** 1 Myeloma Center, Winthrop P. Rockefeller Institute, Department of Internal Medicine and; 2 Arkansas Children’s Nutrition Center, University of Arkansas for Medical Sciences (UAMS), Little Rock, Arkansas, USA.

**Keywords:** Hematology, Oncology, Bone disease, Cancer immunotherapy

## Abstract

We have previously demonstrated that cystatin E/M (CST6), which is elevated in a subset of patients with multiple myeloma (MM) lacking osteolytic lesions (OLs), suppresses MM bone disease by blocking osteoclast differentiation and function. CST6 is a secreted type 2 cystatin, a cysteine protease inhibitor that regulates lysosomal cysteine proteases and the asparaginyl endopeptidase legumain. Here, we developed B cell maturation antigen (BCMA) CST6 chimeric antigen receptor T cells (CAR-T cells), which lysed MM cells and released CST6 proteins. Our in vitro studies show that these CAR-T cells suppressed the differentiation and formation of tartrate-resistant acid phosphatase–positive (TRAP^+^) osteoclasts. Using xenografted MM mice, bioluminescence images showed that both BCMA–CAR-T and BCMA–CST6–CAR-T cells inhibited MM growth to a similar extent. Reconstructed micro–computed tomography images revealed that BCMA–CST6–CAR-T cells, but not BCMA–CAR-T cells, prevented MM-induced bone damage and decreased osteoclast numbers. Our results provide a CAR-T strategy that targets tumor cells directly and delivers an inhibitor of bone resorption.

## Abstract 2

BCMA-CST6-CAR-T cells target multiple myeloma and release large amounts of CST6 to suppress osteolytic lesions.

## Graphical abstract

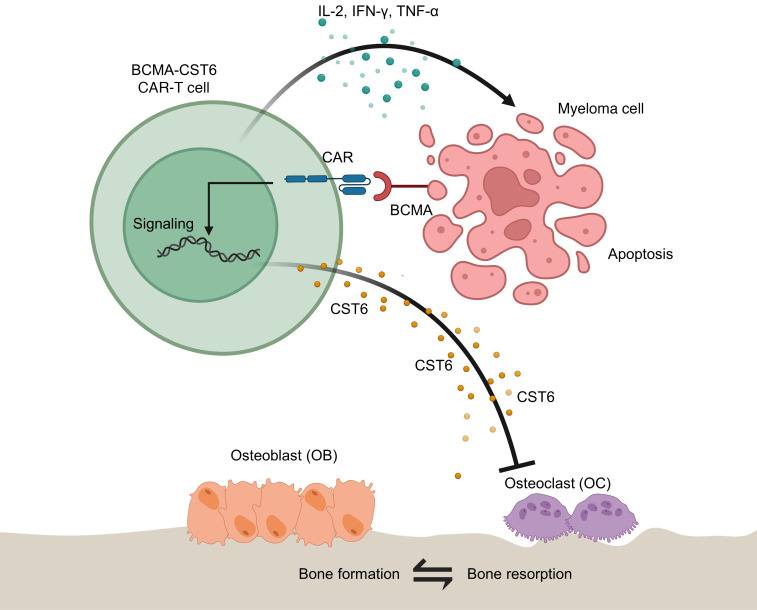

## Introduction

Bone homeostasis is maintained by the coupled activity of bone-forming osteoblasts and bone-resorbing osteoclasts (1). Osteolytic lesions are a hallmark of multiple myeloma (MM), occurring at sites of large focal tumor growth (2). This uncoupling process is driven by MM-derived secreted molecules that modulate the local bone microenvironment, such as RANKL (3), parathyroid hormone–related peptide (PTHrP) (4), and Dickkopf 1 (DKK1) (5). RANKL and PTHrP stimulate osteoclast formation and activity, leading to bone resorption, whereas DKK1 inhibits osteoblasts and thus prevents bone repair (6, 7). The growth factors TGF-β and insulin-like growth factor 1 (IGF-1), released from the bone matrix, together with osteoclast and mesenchymal stem cell–produced IL-6, stimulate MM cell growth and survival (8, 9). Moreover, the interaction between osteoclasts and MM cells accelerates bone resorption. This phenomenon results in an elevation of serum calcium concentration, thereby increasing the risk of hypercalcemia (10).

Cystatin E/M (CST6), a secretory protein with a molecular weight of approximately 14–17 kDa, belongs to the type 2 cystatin family, which encompasses cysteine proteinase inhibitors responsible for modulating lysosomal cysteine proteases as well as the asparaginyl endopeptidase legumain (LGMN). Our investigations have demonstrated that both purified CST6 and bone marrow serum from patients with MM who have elevated CST6 expression exert a CST6-dependent inhibition of osteoclast differentiation and function (11). Although CST6 substantially inhibited the activity of osteoclast-specific protease cathepsin K and prevented osteoclast differentiation and function, recombinant CST6 proteins showed a short serum half-life (*t_1/2_*) in a mouse model (11). As a therapeutic agent, CST6 protein should be maintained consistently at relatively high levels.

B cell maturation antigen–specific (BCMA-specific) chimeric antigen receptor T cell (CAR-T cell) therapies are associated with high response rates in relapsed/refractory MM (RRMM) (12, 13). Importantly, the FDA has approved anti–BCMA-specific CAR-T cell therapies for the treatment of MM (14–16). With the advancement of technology, researchers are also enhancing the functionalities of CAR-T cells. Fourth-generation CAR-T cells, also known as armored CAR-T cells, release a transgenic protein upon CAR engagement of their cognate antigen (17). Fourth-generation CAR-T cells are thereby used as “living factories” to produce and deposit substances only in the targeted tissue (18). CAR-T cells engineered to secrete IL-12 have exhibited augmented activity and a sustained presence in diverse hematologic and solid tumor models. This includes heightened resilience against Treg-mediated inhibition in vitro and intensified cytotoxicity (19–21). Moreover, the generation of IL-18 by CAR-T cells has been demonstrated to result in enhanced antitumor efficacy and modifications in the tumor microenvironment (17, 22).

Because CST6 is a secretory protein, fourth-generation CARs should provide an ideal approach to deliver this protein around nascent focal lesions lasting for a long period and at a stable concentration. Here, we demonstrate that fourth-generation BCMA–CST6–CAR-T cells were able to simultaneously ablate MM cells and suppress osteolytic lesions.

## Results

### Development and characterization of the fourth generation of BCMA–CST6–CAR-T cells.

**Figure 1:**
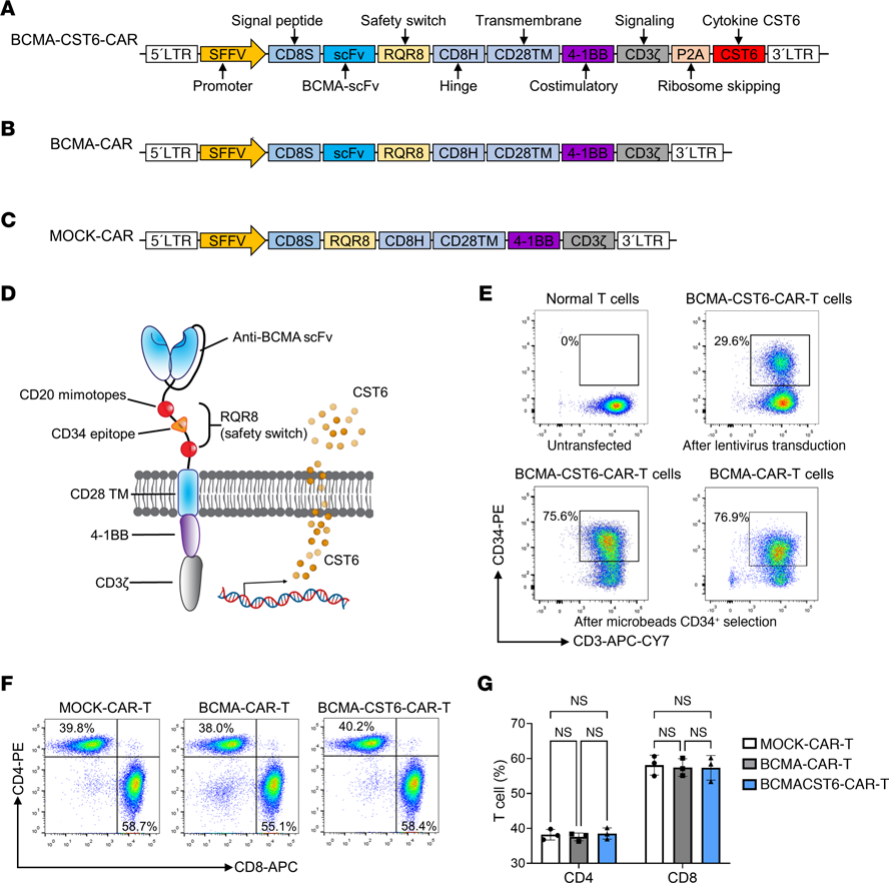
Construction and characteristics of BCMA–CST6–CAR-T cells. (**A**) Generation of BCMA-CST6-CAR constructs. The BCMA-CST6-CAR vector was constructed by BCMA-specific scFvs, a safety switch (RQR8) in the hinge region, and a 4-1BB coactivation domain with CD3ζ. A P2A self-cleaving peptide was inserted between the CAR vectors and CST6. (**B**) The BCMA-CAR vector contained BCMA-scFv, a safety switch, and a 4-1BB coactivation domain with CD3ζ. (**C**) The MOCK-CAR vector only contained a safety switch and a 4-1BB coactivation domain with CD3ζ. All vectors contained SFFV promoters to drive expression of targeted gene(s) in CAR T cells. (**D**) Schematic representation of the BCMA–CST6–CAR-T structure. (**E**) Lentiviruses combined with the RetroNectin transfection technology were engineered with CD3^+^ T cells derived from healthy donor PBMCs. CAR-T cells were detected with RQR8-specific anti-CD34 antibodies by flow cytometry. CD34^+^ rates were 29.6% for BCMA–CST6–CAR-T cells. CD34^+^ cells were enriched to 75.6% for BCMA–CST6–CAR-T cells sorted with anti-CD34 microbeads. CD34^+^ cells were enriched to 76.9% for sorted BCMA–CAR-T cells. (**F**) Flow cytometric analyses revealed CD4^+^/CD8^+^ ratios of MOCK–CAR-T cells, BCMA–CAR-T cells, and BCMA–CST6–CAR-T cells (*n* = 3, representative result from 3 independent experiments). (**G**) Bar plots represent the frequency of CAR-T cells gated on CD4^+^ and CD8^+^ T cells (*n* = 3). Data represent the mean ± SD. One-way ANOVA was used for the statistical analysis; NS = *P* > 0.05. LTR, long terminal repeat.

We constructed a fourth-generation BCMA-CST6-CAR vector by BCMA-specific, single-chain variable fragments (scFvs) (derived from clone C11D5.3) (23, 24), a safety switch (marker-suicide gene RQR8) (25), in the hinge region, and a 4-1BB coactivation domain with CD3ζ. A P2A self-cleaving peptide was inserted between CAR and CST6 (Figure 1, A and D). To decrease the risk of severe immunological side effects, we integrated RQR8 with 2 CD20 mimotopes as a suicide molecule and 1 CD34 epitope for detection and positive selection. The BCMA-CAR vector contains BCMA-scFv, a safety switch, and a 4-1BB coactivation domain with CD3ζ (Figure 1B). The MOCK-CAR vector only contains a safety switch and a 4-1BB coactivation domain with CD3ζ (Figure 1C). All segments were of human origin.

We used lentiviruses combined with the RetroNectin transfection technology to modify CD3^+^ T cells from healthy donor PBMCs (26). The transfection efficiency of CAR-T cells was detected by flow cytometry using the anti–inserted RQR8–specific CD34 antibody. We found that 29.6% of cells were BCMA-CST6-CAR T cells. Transfected BCMA–CST6–CAR-T cells were isolated to 75.6% purity using anti-CD34 microbeads, and transfected BCMA–CAR-T cells were isolated to 76.9% purity (Figure 1E).

We conducted phenotypic analysis of CAR-T cells using flow cytometry, which included the assessment of CD4 and CD8 expression ratios. The mean percentage of CD4^+^ BCMA–CST6–CAR-T cells was 38.5%, while 57.3% were CD8^+^. This ratio showed no statistically significant differences compared with the BCMA–CAR-T cells (37.6% CD4^+^ and 57.4% CD8^+^) cells and MOCK–CAR-T cells (38.2% CD4^+^ and 58.1% CD8^+^) (Figure 1, F and G).

### BCMA–CST6–CAR-T cells bind specifically to human BCMA in vitro.

**Figure 2:**
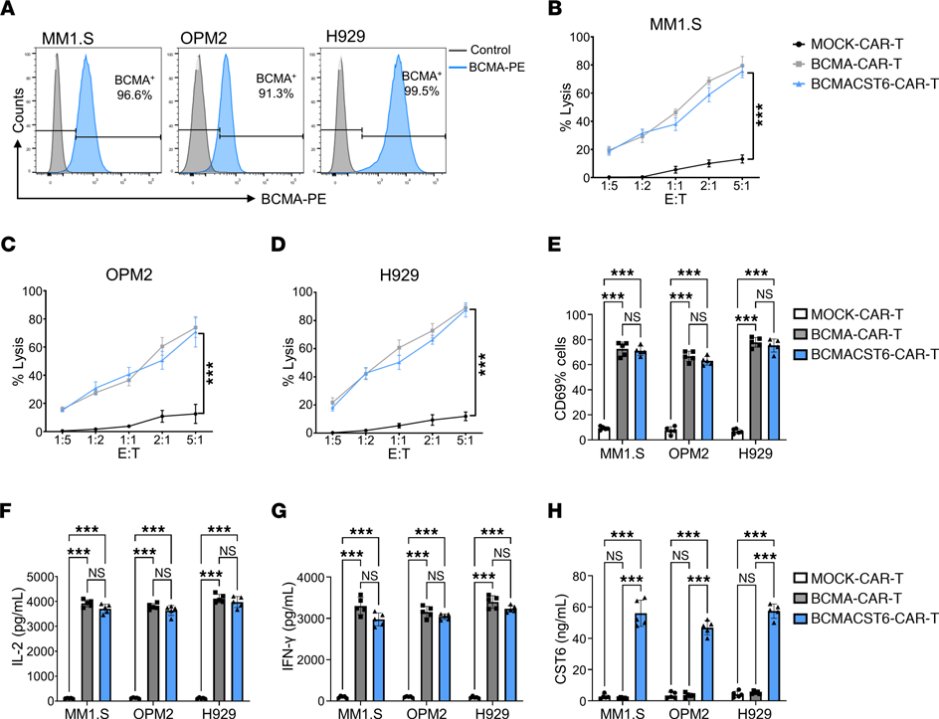
BCMA–CST6–CAR-T cells efficiently kill MM cell lines and release cytokines in vitro. (**A**) Flow cytometric analysis of BCMA expression in MM1.S, OPM2, and H929 cell lines. BCMA was highly expressed in MM1.S, OPM2, and H929 cell lines. (**B**–**D**) In vitro cytolytic activity of BCMA–CST6–CAR-T cells. CAR-T cells were added to MM1.S (**B**), OMP2 (**C**), and H929 (**D**) cell lines at an E/T ratio of 1:5 to 5:1. After 24 hours of coculturing, cytolytic activity was measured. Percentage of  lysis = (experimental lysis − spontaneous lysis)/(maximal lysis − spontaneous lysis) × 100%. *n* = 5. (**E**) Expression of the T cell activation marker CD69 was detected at an E/T ratio of 5:1 after 24 hours of coculturing (*n* = 5). (**F**) IL-2 concentrations in supernatants were detected at an E/T ratio of 5:1 after 24 hours of coculturing (*n* = 5). (**G**) IFN-γ concentrations in supernatants were detected at an E/T ratio of 5:1 after 24 hours of coculturing (*n* = 5). (**H**) CST6 concentrations in supernatants were detected at an E/T ratio of 5:1 after 24 hours of coculturing (*n* = 5). Data represent the mean ± SD. ***P < 0.001, by 1-way ANOVA; NS = *P* > 0.05.

We detected BCMA expression in the 3 MM cell lines MM1.S (96.6% cells were BCMA^+^), OPM2 (91.3% cells were BCMA^+^), and H929 (99.5% cells were BCMA^+^) (Figure 2A). We found that BCMA was highly expressed in these 3 MM cell lines. We used these 3 MM cell lines for our in vivo and in vitro experiments.

To further verify the specificity of the BCMA–CST6–CAR-T cells, CAR-T cells were incubated with FITC-labeled human BCMA. After incubation of FITC-labeled human BCMA, both BCMA–CAR-T cells and BCMA–CST6–CAR-T cells expressed a strong and equivalent FITC signal. In contrast, we detected no FITC signal in the MOCK–CAR-T cells (Supplemental Figure 1, A and B; supplemental material available online with this article; https://doi.org/10.1172/JCI171396DS1). These results indicate that BCMA–CST6–CAR-T cells and BCMA–CAR-T cells specifically bound human BCMA with similar affinity.

### BCMA–CST6–CAR-T cells show efficient cytotoxicity of MM cell lines and cytokine release in vitro.

CAR-T cells were incubated with human MM cell lines at different ratios. Analysis of coculture-killing assays of CAR-T cells with MM1.S, OPM2, and H929 indicated that effector CAR-T cell–mediated target tumor cytotoxicity proportionally increased with the effector-to-target (E/T) ratio. BCMA–CST6–CAR-T cells incubated with MM1.S-mCherry cells at a ratio of 5:1 revealed that the percentage of MM1.S-mCherry fluorescence–positive cells lysed after 24 hours of coculturing was 75.5%. MM1.S cell lysis increased 5.7-fold in the BCMA–CST6–CAR-T group compared with MM1.S cells in the MOCK–CAR-T group. We observed no significant difference in killing between the BCMA–CAR-T group and the BCMA-CST6-CAR-T group (Figure 2B). The percentage of target cells lysed was 70.8% in the OPM2 cell line at an E/T ratio of 5:1, and there was a 5.6-fold increase in the percentage of lysis seen with BCMA–CST6–CAR-T cells compared with the average of MOCK–CAR-T cells (Figure 2C). Similarly, the percentage of target cells lysed was 87.6% in the H929 cell line, and there was a 7.3-fold increase in the percentage of lysis seen with BCMA–CST6–CAR-T cells compared with the average of MOCK–CAR-T cells (Figure 2D).

To assess CAR-T cell activation, we measured the expression of the T cell activation marker CD69 in CAR-T cells after 24 hours of coculturing with MM1.S, OPM2, or H929 cells. We found that CD69 expression was significantly increased in both BCMA–CST6–CAR-T (71.0%) and BCMA–CAR-T (72.5%) cell groups compared with the MOCK–CAR-T cell group (8.7%) (*P* < 0.001) when cocultured with MM1.S cells (Figure 2E). Activation of BCMA–CST6–CAR-T cells and BCMA–CAR-T cells was also confirmed by increased CD69 expression when these cells were cocultured with OPM2 or H929 cells (Figure 2E). ELISA showed that expression of both IL-2 and IFN-γ cytokines was significantly increased in the BCMA–CST6–CAR-T and BCMA–CAR-T cell groups compared with the MOCK–CAR-T cell group (Figure 2, F and G; *P* < 0.001).

To measure the level of CST6 secretion from BCMA–CST6–CAR-T cells, BCMA–CST6–CAR-T cells were incubated with MM cells at a ratio of 5:1, and at different time points, CST6 protein levels in culture media were measured by ELISA. As the duration of coculturing of BCMA–CST6–CAR-T cells and MM cells was extended, the concentration of CST6 in the culture medium increased accordingly. By 24 hours, the concentration reached a plateau. There was a slight increase at 36 hours and 48 hours, but we observed no significant differences compared with the 24-hour culturing period (Supplemental Figure 1C). When cocultured with MM1.S cells for 24 hours, CST6 was detected at 56.0 ng/mL in the BCMA-CST6-CAR-T group, which was a significantly higher concentration than that detected in the MOCK-CAR-T group (2.7 ng/mL) or the BCMA–CAR-T group (1.9 ng/mL). This phenomenon was consistent when these CAR-T cells were cocultured with OPM2 or H929 cells for 24 hours (Figure 2H).

### BCMA–CST6–CAR-T cells kill primary human MM cells efficiently in vitro.

**Figure 3:**
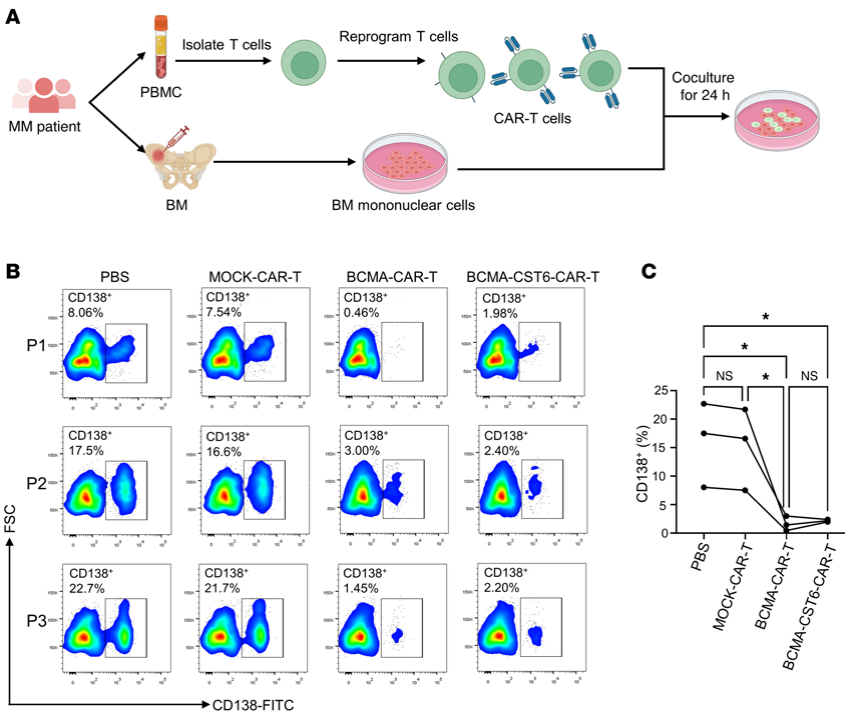
BCMA–CST6–CAR-T cells selectively kill primary human MM cells in vitro. (**A**) Experimental workflow for the process of CAR-T cell–mediated cytotoxicity against primary human MM cells. Samples from 3 patients were collected. CAR-T cells were generated using the donors’ peripheral blood T cells and directed against their own BMMCs. (**B**) Flow cytometric analysis was used to identify primary human CD138^+^ MM cells from 3 patient samples with the following different treatments for 24 hours: PBS, MOCK–CAR-T cells, BCMA–CAR-T cells, and BCMA–CST6–CAR-T cells. (**C**) Percentage of the subpopulation of human CD138^+^ MM cells decreased in 3 of 3 primary MM samples after BCMA–CST6–CAR-T treatment. *P < 0.05, by 1-way ANOVA; NS = *P* > 0.05.

In addition to generating CAR-T cells using PBMCs from healthy donors, we have demonstrated that tumor lysis occurred using CAR-T cells generated from MM patients’ PBMCs when cocultured with their corresponding CD138^+^ MM cells in the bone marrow. We collected samples from 3 patients. CAR-T cells were generated using T cells isolated from the peripheral blood and directed against their own tumor cells (Figure 3A). Despite the fact that each of the 3 patients with MM had distinct percentages of CD138^+^ MM cells in bone marrow mononuclear cells (BMMCs), BCMA–CST6–CAR-T cell treatment significantly decreased the percentage of CD138^+^ cells in BMMC samples from all MM patients (Figure 3, B and C; *P* < 0.05). These results show that primary MM cells were effectively lysed by BCMA–CST6–CAR-T cells.

Overall, these results indicate that BCMA–CST6–CAR-T cells were strongly activated after exposure to BCMA^+^ MM cell lines and that CST6 secretion did not affect the activation or function of BCMA–CAR-T cells.

### BCMA–CST6–CAR-T cells suppress the formation of tartrate-resistant acid phosphatase–positive osteoclasts.

**Figure 4:**
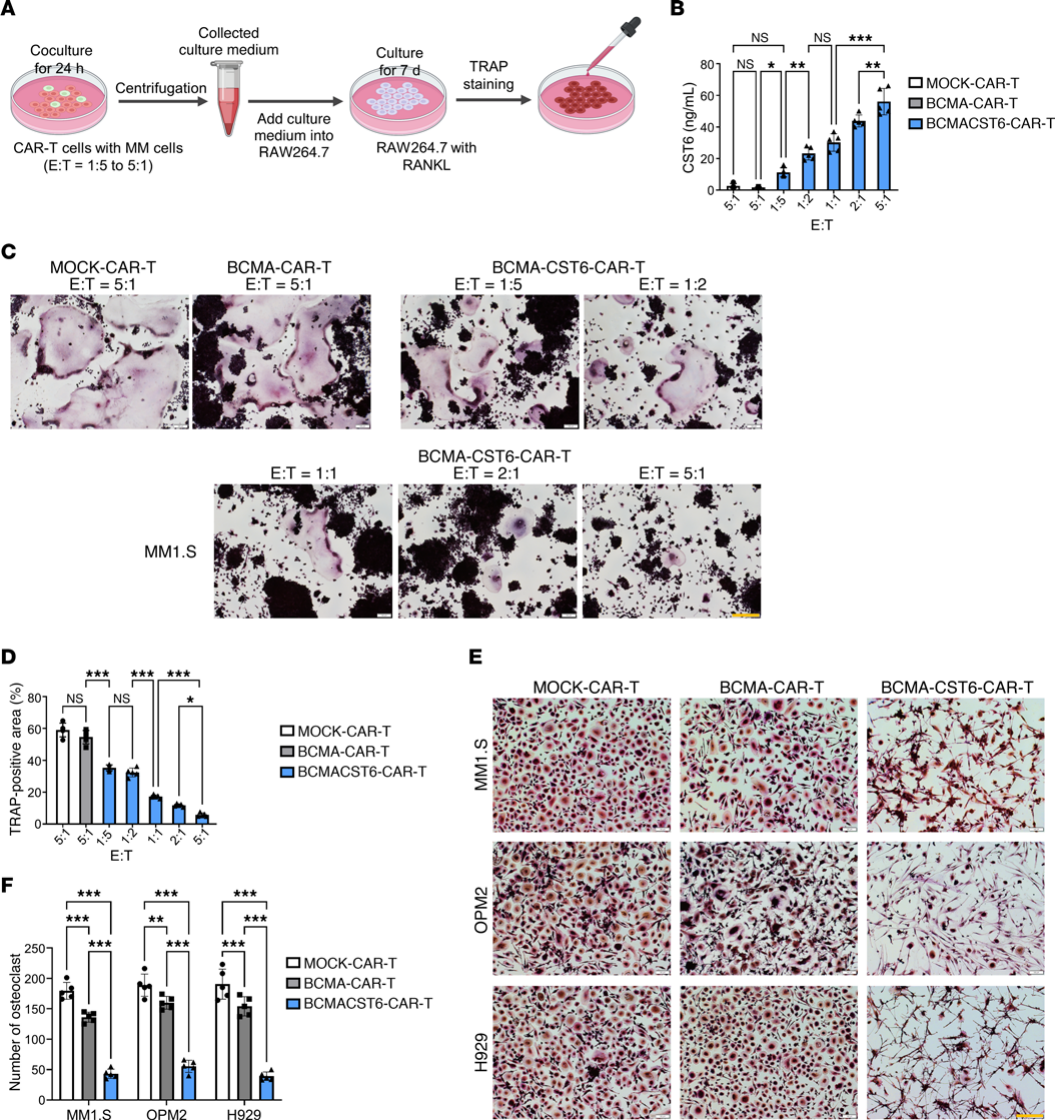
BCMA–CST6–CAR-T cells suppress osteoclast differentiation. (**A**) Experimental workflow for the detection of TRAP^+^ osteoclasts. (**B**) CST6 concentrations in supernatants were detected at E/T ratios of 1:5 to 5:1 after 24 hours of coculturing (*n* = 5). (**C**) CAR-T cells were incubated with MM1.S cells at ratios of 1:5 to 5:1 for 24 hours, and CM were collected and added into RAW 264.7 cells with RANKL. On day 7, osteoclasts were stained with TRAP solution (*n* = 5, representative result from 5 independent experiments). Scale bars: 200 μm. (**D**) Bar plots present quantifications of the TRAP^+^ area (*n* = 5). (**E**) Human CD14^+^ monocytes sorted from bone marrow of patients with MM were differentiated into osteoclasts with M-CSF and RANKL for 7 days. CM were added to human CD14^+^ monocyte culture media (50%) for another 7 days. On day 14, osteoclasts were stained with TRAP solution (*n* = 5, representative result from 5 independent experiments). Scale bars: 200 μm. (**F**) Bar plots represent the number of TRAP^+^ osteoclasts derived from human CD14^+^ monocytes per view (*n* = 5). Data represent the mean ± SD. *P < 0.05, **P < 0.01, and ***P < 0.001, by 1-way ANOVA; NS = *P* > 0.05.

RAW 264.7 cells are monocyte/macrophage-like cells that can differentiate into osteoclasts upon exposure to RANKL (27). To determine whether BCMA–CST6–CAR-T–derived CST6 could suppress osteoclast differentiation, conditioned media (CM) from 24-hour cocultures of BCMA–CST6–CAR-T cells and MM cells at various ratios were collected and added to the culture media (50%) with RANKL and RAW 264.7 cells and cultured for 7 days (Figure 4A). With the increased E/T ratios in MM1.S cells, the concentration of CST6 in the CM also increased (Figure 4B). As the concentration of CST6 in the CM increased, we noted a significant reduction in the tartrate-resistant acid phosphatase–positive (TRAP^+^) areas of osteoclasts (Figure 4, C and D). This phenomenon remained consistent when using OPM2 or H929 cells (Supplemental Figure 2).

CM from cultures of CAR-T cells with MM1.S, OPM2, or H929 cells at a ratio of 5:1 for 24 hours were collected and added to human osteoclast progenitor and CD14^+^ monocyte culture media (50%) along with RANKL to induce osteoclast differentiation. Cells were cultured for 7 days. CM from BCMA-CST6-CAR-T cells significantly suppressed the formation of TRAP^+^ osteoclasts (Figure 4, E and F).

### BCMA–CST6–CAR-T cells suppress MM growth in vivo.

**Figure 5:**
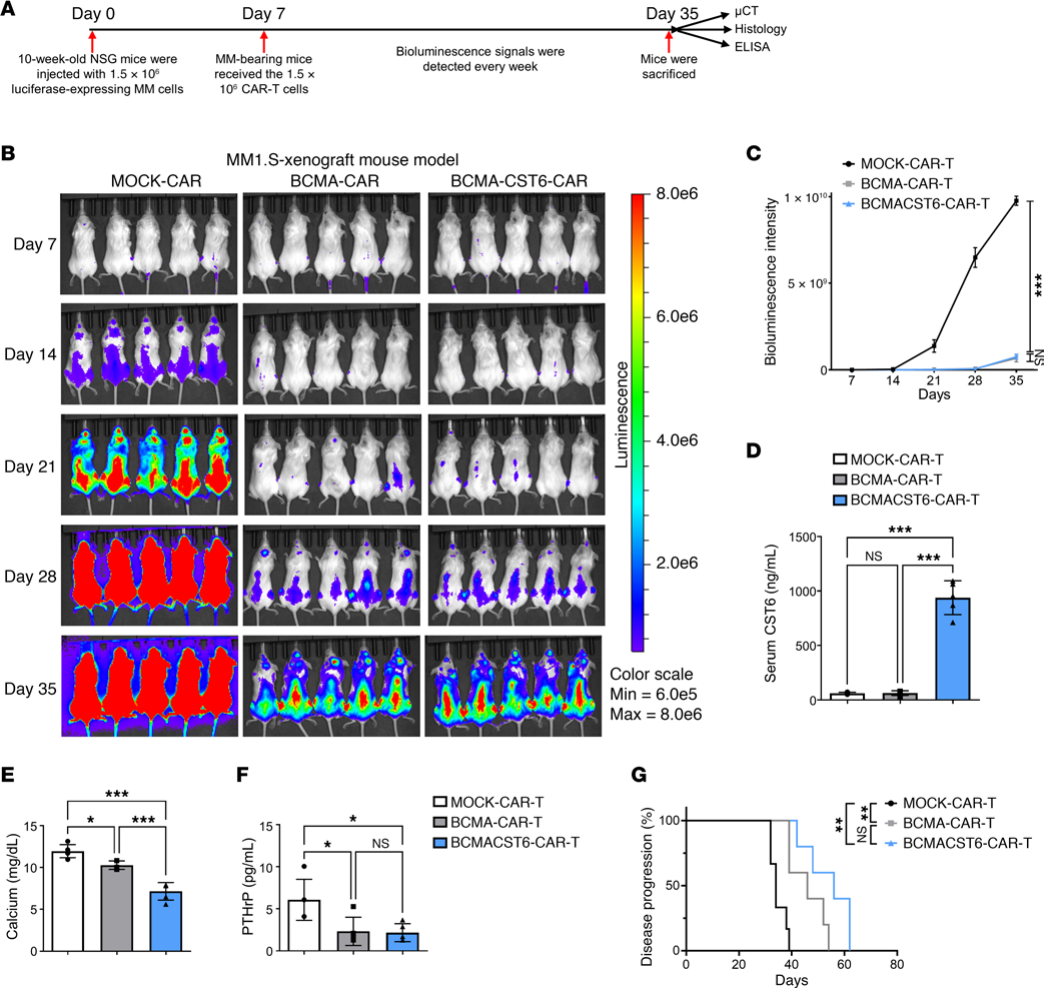
BCMA–CST6–CAR-T cells suppress MM1.S cell growth in vivo. (**A**) Schematic of the experimental plan. NSG mice were administered 1.5 × 10^6^ MM cells via i.v. injection. On day seven, 1.5 × 10^6^ CAR-T cells were administered after injection of MM cells. Myeloma progression was monitored until the mice developed hind limb paralysis. (**B**) Tumor burden was evaluated by bioluminescence imaging of MM1.S cell–bearing mice treated with MOCK–CAR-T, BCMA–CAR-T, or BCMA–CST6–CAR-T cells. (**C**) Quantitative analysis of bioluminescence intensity (*n* = 5). (**D**) Mouse serum levels of CST6 protein detected by ELISA (*n* = 5). (**E**) Mouse serum levels of calcium detected by ELISA (*n* = 5). (**F**) Mouse serum levels of PTHrP detected by ELISA (*n* = 5). (**G**) Kaplan-Meier disease progression analysis of CAR-T treatment in NSG models (*n* = 5). Data represent the mean ± SD. *P < 0.05, **P < 0.01, and ***P < 0.001, by 1-way ANOVA (**C**–**F**) and log-rank test (**G**); NS = *P* > 0.05.

To evaluate the in vivo anti-tumor activity and anti–bone resorption of BCMA–CST6–CAR-T cells, we injected MM1.S, OPM2, or H929 cells expressing luciferase into NOD.Cg-*Prkdc^scid^ Il2rg^tm1Wjl^*/SzJ (NSG) mice. On day 7, MM-bearing mice received CAR-T cells. Luciferase signals were measured every 7 days. When we sacrificed mice on day 35, we evaluated the CST6 concentration in mouse sera, and mouse tibiae were collected for micro-CT (μCT) and TRAP staining (Figure 5A). Bioluminescence imaging of MM-bearing mice revealed the BCMA–CST6–CAR-T cells were effective in terms of antitumor activity, yielding near-complete tumor clearance by day 21, whereas myeloma cells continued to grow in the MOCK–CAR-T group. The BCMA–CST6–CAR-T and BCAM–CAR-T cells had very similar tumor growth–inhibiting effects (Figure 5B). This observation was supported by measurements of tumor burden shown in the bioluminescence intensity curve (Figure 5C).

In the MM1.S xenograft model, the CST6 serum concentration was significantly higher in the BCAM–CST6–CAR-T group than in the MOCK– or BCMA–CAR-T cell groups (938.4 vs. 60.9 and 64.2 ng/mL; *P* < 0.001) (Figure 5D). These results demonstrate that BCMA–CST6–CAR-T cells not only eliminated tumor cells but also secreted CST6 in vivo.

Furthermore, we also assessed the concentration of calcium and PTHrP in the serum. In the MM1.S xenograft model, the serum calcium concentration was significantly decreased in the BCAM–CST6–CAR-T group compared with the MOCK- and BCMA–CAR-T groups (7.15 vs. 11.95 and 10.27 mg/dL; *P* < 0.001) (Figure 5E). The serum PTHrP concentration in the BCAM–CST6–CAR-T group was significantly decreased compared with that in the MOCK–CAR-T group, but with no significant differences compared with the BCMA–CAR-T group in the MM1.S xenograft model (Figure 5F).

We repeated the in vivo experiment to monitor the occurrence of paralysis in mice. Mice were sacrificed and recorded as having had disease progression when they developed paralysis or extreme weakness. Mice in the BCMA–CST6–CAR-T group took significantly longer to develop paralysis. The time to disease progression for the BCMA–CST6–CAR-T group (median disease progression = 56 days) was longer than that for the BCMA–CAR-T group (median disease progression = 46 days) (*P* = 0.0777) (Figure 5G).

**Figure 6:**
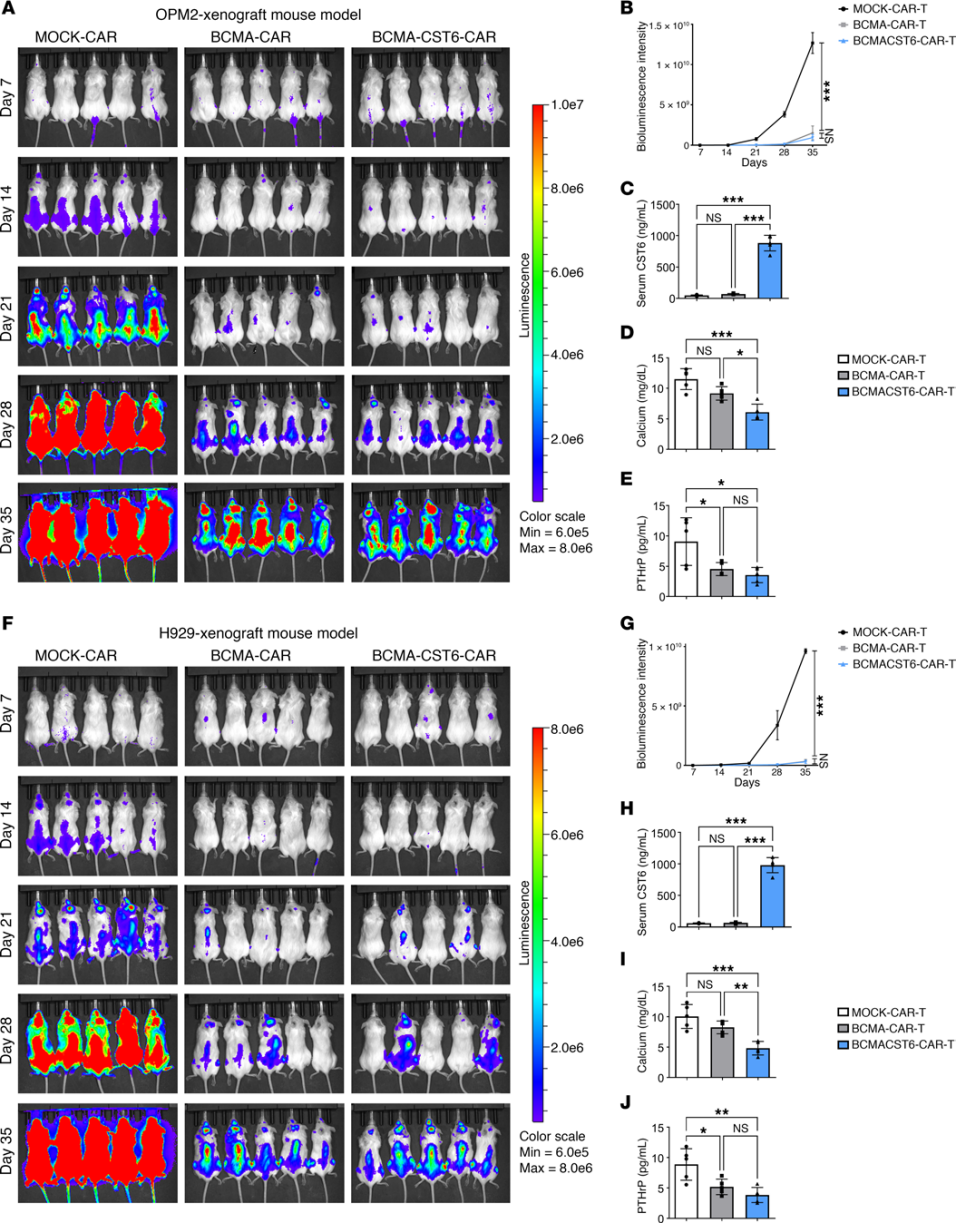
Validation of BCMA–CST6–CAR-T cell suppression of MM growth in OPM2 and H929 MM cell lines in vivo. (**A**) Tumor burden was evaluated by bioluminescence imaging of OPM2 cell–bearing mice treated with MOCK–CAR-T cells, BCMA–CAR-T cells, or BCMA–CST6–CAR-T cells. (**B**) Quantitative analysis of bioluminescence intensity in the OPM2 xenograft model (*n* = 5). (**C**–**E**) Mouse serum levels of CST6 (**C**), calcium (**D**), and PTHrP (**E**) detected by ELISA in the OPM2 xenograft model (*n* = 5). (**F**) Tumor burden was evaluated by bioluminescence imaging of H929 cell–bearing mice treated with MOCK–CAR-T cells, BCMA–CAR-T cells, or BCMA–CST6–CAR-T cells (*n* = 5). (**G**) Quantitative analysis of bioluminescence intensity in the H929 xenograft model (*n* = 5). (**H**–**J**) Mouse serum levels of CST6 (**H**), calcium (**I**), and PTHrP (**J**) detected by ELISA in the H929 xenograft model (*n* = 5). Data represent the mean ± SD. *P < 0.05, **P < 0.01, and ***P < 0.001, by 1-way ANOVA; NS = *P* > 0.05.

In the OPM2 xenograft model, BCMA–CST6–CAR-T cells also demonstrated effectiveness in antitumor activity (Figure 6, A and B). The BCAM–CST6–CAR-T group showed the highest CST6 serum concentration and the lowest serum calcium concentration in the OPM2-bearing mice (Figure 6, C and D). Meanwhile, in the OPM2 xenograft model, there were no significant differences in serum PTHrP concentrations between the BCMA–CST6–CAR-T group and the BCMA–CAR-T group (Figure 6E). In the H929 xenograft model, BCMA–CST6–CAR-T cells exhibited antitumor activity (Figure 6, F and G) and effects on serum indicators (Figure 6, H–J) comparable to those observed in the OPM2 xenograft model.

### BCMA–CST6–CAR-T cells inhibit MM cell–induced bone resorption in vivo.

**Figure 7:**
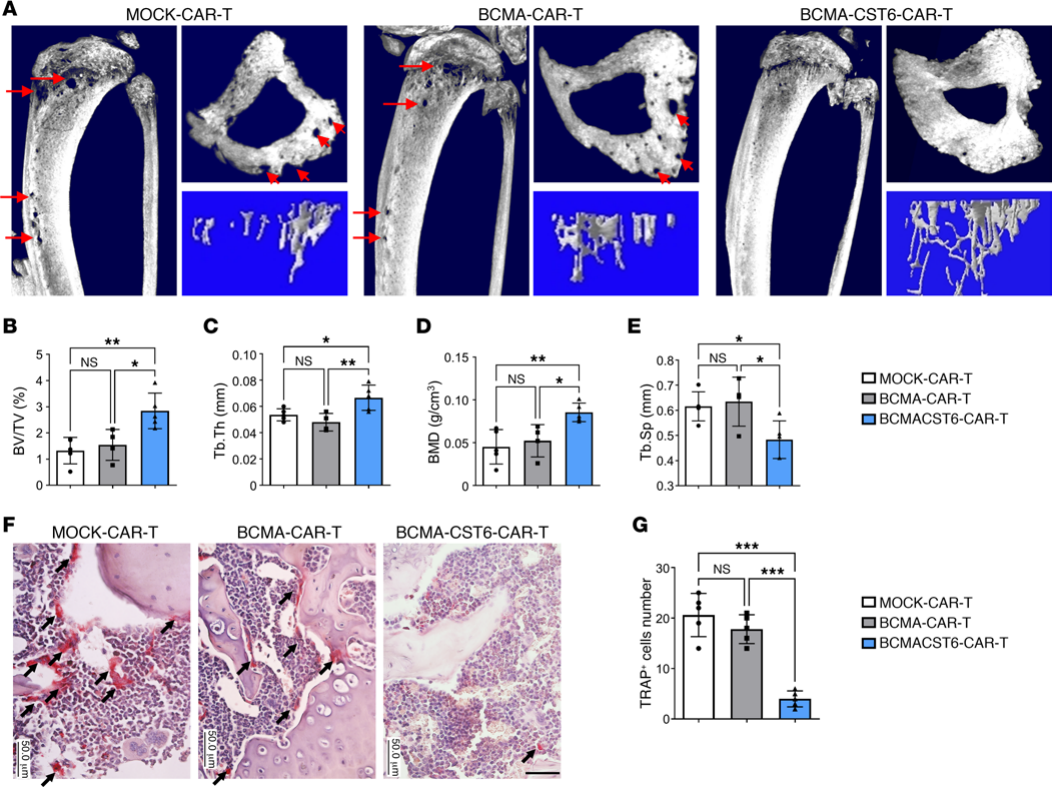
BCMA–CST6–CAR-T cells inhibit MM cell–induced bone resorption in vivo. (**A**) Reconstructed μCT images of tibia sagittal sections show bone lytic lesions (indicated with arrows) and trabecular architecture in the MM1.S xenograft model (*n* = 5, representative result from 5 mice). (**B**–**E**) Number of bone lytic lesions on the right medial tibia surface according to the trabecular bone parameters BV/TV (**B**); Tb.Th (**C**); BMD (**D**); and Tb.Sp (**E**) in the MM1.S xenograft model (*n* = 5). (**F**) TRAP staining showed osteoclasts (indicated with arrows) in tibias derived from the MM1.S xenograft model (*n* = 5, representative result from 5 mice). Scale bar: 50 μm. (**G**) Histomorphometric analyses of the number of TRAP-stained osteoclasts per view (*n* = 5). Data represent the mean ± SD. *P < 0.05, **P < 0.01, and ***P < 0.001, by 1-way ANOVA; NS = *P* > 0.05.

Reconstructed μCT images of mouse tibiae showed that BCMA–CST6–CAR-T cells significantly suppressed osteolytic lesions in MM-bearing mice (Figure 7A and Supplemental Figure 3, A and C). BCMA–CST6–CAR-T cells showed substantially increased trabecular bone volume over total volume (BV/TV), trabecular thickness (Tb.Th), and bone mineral density (BMD) and decreased trabecular separation (Tb.Sp) in the BCMA–CST6–CAR-T cell–treated mice compared with those in the BCMA–CAR-T cell–treated mice (Figure 7, B–E, and Supplemental Figure 3, B and D). TRAP staining of mouse tibia sections demonstrated that the BCMA–CST6–CAR-T cells significantly reduced osteoclast numbers and the proportion of bone surface occupied by osteoclasts in MM-bearing mice (Figure 7, F and G).

## Discussion

MM ranks as the second most common hematologic malignancy, characterized by the development of osteolytic lesions in 80% of patients (28). The hallmark of myeloma bone disease is the excessive activation of osteoclasts (29, 30). MM cells secrete factors, such as RANKL and PTHrP, that promote osteoclastogenesis, driving the activation of osteoclasts and consequent bone lesions (31). Hence, targeting osteoclasts stands as a substantial strategy to disrupt this detrimental osteolytic cycle (32, 33). In our previous study, we found that recombinant CST6 protein effectively suppresses osteoclast differentiation and bone resorption in vitro. Moreover, recombinant CST6 demonstrates the capacity to mitigate bone loss induced by MM cells, as evidenced by an in vivo MM mouse model (11).

BCMA-targeting CAR-T therapy has proven very successful in RRMM therapy (12). FDA-approved BCMA–CAR-T cell therapy has shown impressive overall response rates (14, 16). The median progression-free survival of idecabtagene vicleucel (Ide-Cel; bb2121) was 8.8 months for the entire study and in the highest-dose cohort, the median progression-free survival was 12.1 months (12, 14, 34). However, BCMA–CAR-T did not alleviate the osteolytic lesions. Fourth-generation CARs should be an ideal approach to delivering CST6 around nascent focal lesions. This design effectively addresses the drawback of CST6’s short half-life. Activated CAR-T cells can continuously produce CST6, maintaining it at a high level within the targeted tissue. Here, we engineered fourth-generation BCMA–CST6–CAR-T cells to eliminate MM cells and suppress osteolytic lesions.

The BCMA–CST6–CAR-T CAR is composed of an extracellular scFv (derived from clone C11D5.3) specific for recognizing BCMA. The clone C11D5.3 is also a component of the commercial BCMA–CAR-T product Ide-Cel (23, 24). BCMA–CST6–CAR-T cells displayed superior activity in vitro and in vivo after exposure to BCMA^+^ MM cell lines. We have also demonstrated that cytotoxicity occurred by CAR-T cells generated from patients using their tumors as the target. Importantly, we observed no significant difference in the killing between BCMA–CAR-T cells and BCMA–CST6–CAR-T cells, which means that CST6 secretion did not affect the activation or function of BCMA–CAR-T cells. Simultaneously, we observed that BCMA–CST6–CAR-T cells could release a substantial amount of CST6 protein. The amount of CST6 protein released increased with an escalating E/T ratio in vitro. We attributed this to the higher E/T ratio, which led to an increased number of activated CAR-T cells, generating more CST6 protein. High concentrations of CST6 protein substantially suppressed the formation of TRAP^+^ osteoclasts.

Hypercalcemia is the most common metabolic complication for patients with MM, with excessive osteolysis playing a substantial contributory role in its pathogenesis (10). Because BCMA–CST6–CAR-T cells effectively suppress osteoclast differentiation and bone resorption in vivo, serum calcium levels were significantly decreased in the BCAM–CST6–CAR-T group compared with the MOCK– and BCMA–CAR-T groups. This effect could potentially alleviate hypercalcemia in patients with MM. The dysregulated expression of PTHrP in advanced cancers causes malignancy-associated hypercalcemia (35). The serum PTHrP concentration in the BCAM–CST6–CAR-T group was substantially decreased compared with that in the MOCK–CAR-T group but with no significant differences compared with the BCMA–CAR-T group, confirming that PTHrP levels are determined by tumor burden and not by CST6 (36). In the BCMA–CAR-T and BCAM–CST6–CAR-T groups, the tumor cells were effectively suppressed, leading to a subsequent decrease in serum PTHrP.

**Figure 8:**
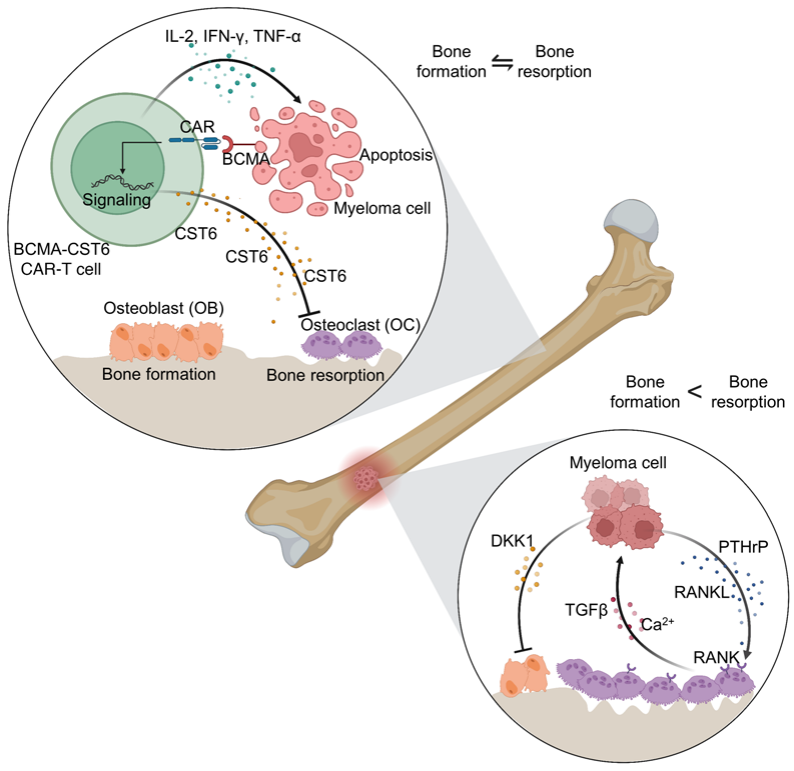
Schematic representation of the role of BCMA–CST6–CAR-T cells in MM growth and focal lesions. BCMA–CST6–CAR-T cells effectively target MM tumor cells in vitro and in vivo and secrete CST6 protein, thereby inhibiting osteoclastogenesis and bone resorption.

In summary, this work presents a rational approach to engineering BCMA–CST6–CAR-T cells that can effectively target MM tumor cells in vitro and in vivo and that can release large amounts of CST6 protein, which significantly suppresses osteolytic lesions in MM (Figure 8). This work provides proof of concept that BCMA–CST6–CAR-T cells represent a multimodality immunotherapy treatment for MM that suppresses osteoclast-driven bone resorption and focal tumor growth.

## Methods

### Patient samples and cell lines.

PBMCs and BMMCs were obtained from the UAMS Myeloma Center Tissue Biorepository and Procurement Core. Human MM1.S cells and mouse RAW264.7 cells were purchased from the American Type Culture Collection (ATCC). Human OPM2 and H929 cells were provided by Siegfried Janz (Medical College of Wisconsin, Milwaukee, Wisconsin, USA). MM1.S, OPM2, and H929 cell lines were cultured in RPMI 1640 medium (Gibco, Thermo Fisher Scientific) containing 10% fetal calf serum (FCS) (Gibco, Thermo Fisher Scientific), 100 U/mL penicillin and 100 μg/mL streptomycin (Gibco, Thermo Fisher Scientific). The RAW264.7 cell line was cultured in DMEM (Gibco, Thermo Fisher Scientific) containing 10% fetal calf serum (FCS) (Gibco, Thermo Fisher Scientific), 100 U/mL penicillin, and 100 μg/mL streptomycin (Gibco, Thermo Fisher Scientific).

### Construction of the CAR vector.

The BCMA-CST6-CAR vector contained BCMA-scFv (derived from clone C11D5.3) (23, 24), a safety switch RQR8 (25), and a 4-1BB coactivation domain with CD3ζ. A P2A self-cleaving peptide was inserted between CAR and CST6. The BCMA-CAR vector contained BCMA-scFv, a safety switch, and a 4-1BB coactivation domain with CD3ζ. The MOCK-CAR vector only contained a safety switch and a 4-1BB coactivation domain with CD3ζ (Figure 1, A–C). All vectors contained SFFV promoters to drive the expression of CARs.

### Transfection of T cells.

Density-gradient centrifugation was used to isolate PBMCs from healthy donor samples or patients with MM by Ficoll-Paque (General Electric). T cells were isolated from PBMCs with anti-CD3 microbeads (Miltenyi Biotec, 130-050-101) and were cultured in AIM V Medium (Thermo Fisher Scientific) with 5% human AB serum (MilliporeSigma) and 400 IU IL-2 (R&D Systems). Dynabeads of human T-activator CD3/CD28 (Thermo Fisher Scientific, 11161D) were added for T cell expansion and activation (26). T cells were activated for 2–3 days prior to transduction.

Lentivirus particles were used to transduce T cells. T cells and concentrated lentiviruses were added into RetroNectin-precoated plates (Takara Bio) (26). Cells were cultured in AIM V Media for 24 hours, and the transduction step was repeated. After 24 hours, cells were washed with PBS and cultured in fresh media for 7 days. CAR-T cells were detected by BD FACSVerse flow cytometry with anti-CD34 antibodies (BioLegend, clone: 561, no. 343606), anti-CD3 antibodies (BioLegend, clone: HIT3a, no. 300318), and CD34 microbeads (Miltenyi Biotec, 130-046-703) for isolation. CD4 or CD8 phenotypes of CAR-T cells were detected by flow cytometry using anti-CD4 antibodies (BioLegend, clone: RPA-T4, no. 300508) and anti-CD8 antibodies (BioLegend, clone: SK1, no. 344722).

### Specificity assay of BCMA–CST6–CAR-T cells.

To verify the specificity of BCMA–CST6–CAR-T cells, flow cytometry was performed. FITC-labeled human BCMA (AcroBiosystems, BCA-H522y) was generated using the FluoroTag FITC Conjugation Kit (MilliporeSigma, FITC1). Solute FITC and proteins were mixed at a molar ratio of 5:1 and incubated for 2 hours at room temperature in a reaction vial with gentle stirring in a dark room. Then, the conjugated product was purified by ultrafiltration to remove the unconjugated FITC molecule. BCMA–CST6–CAR-T cells (2 × 10^5^) were stained with 100 μL of 3 μg/mL FITC-labeled proteins and anti–human CD3-APC-CY7 antibodies (BioLegend, clone: HIT3a, no. 300318). FITC and APC-CY7 signals were tested by flow cytometry. FITC signal was used to evaluate the binding activity of BCMA–CST6–CAR-T cells.

### Cytotoxicity assay of CAR-T cells.

The cytolytic activity of CAR-T cells was detected on the basis of fluorescence intensity. MM1.S-mCherry cells (20 × 10^3^), OPM2-mCherry cells, or H929-mCherry cells were seeded in 96-well plates. CAR-T cells were added at E/T ratios of 1:5 to 5:1. Fluorescence intensity was measured at excitation 585 nm/emission 620 nm with a plate reader (BioTek) after 24 hours of coculturing. 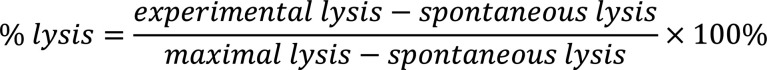


The T cell activation marker CD69 was detected using anti-CD69 antibodies (BioLegend, clone: FN50, no. 310906), and IFN-γ and IL-2 cytokines were measured using ELISA kits (R&D Systems, DIF50C and D2050) and were produced by activated CAR-T cells in supernatants of CAR-T and MM cell cocultures after 24 hours.

### Sandwich ELISA for detection of CST6 protein expression levels.

ELISA plates (BioLegend) were coated with 100 ng monoclonal CST6 antibodies (R&D Systems, clone: 211515, no. MAB1286) in ELISA coating buffer overnight at 4°C. Plates were washed and blocked with 1% BSA (200 μL/well) at 37°C for 1 hour. Plates were washed with PBS. Recombinant CST6 protein (R&D Systems) was used to establish the standard curve (0–100 ng/mL in PBS). CAR-T and MM cell coculture media or mouse serum samples were added to each well and incubated at 37°C for 2 hours. Plates were washed with PBST, followed by incubation with biotinylated polyclonal anti-CST6 antibody (50 μL/well, 0.2 μg/mL in PBS, pH 7.2) (R&D Systems, BAF1286) at 37°C for 2 hours. Plates were washed with PBST. Next, each well was incubated with 50 μL of a 1:10,000 dilution of streptavidin-HRP (Thermo Fisher Scientific) at 37°C for 1 hour. Color development was achieved with addition of substrate (R&D Systems) according to the manufacturer’s instructions, and the reaction was stopped by treatment of the plates with 2 M sulfuric acid (50 μL/well, 0.5 mol/L). The absorbance values were measured at 450 nm. The ELISA results were normalized to total protein concentrations.

### Osteoclast TRAP staining in vitro.

MM1.S cells (20 × 10^3^), OPM2 cells, and H929 cells were seeded, and CAR-T cells were added at an E/T ratio of 1:5 to 5:1, respectively, for 24 hours in each well of 96-well plates. Cocultured CM were collected and added to the culture media (50%) with 50 ng/mL RANKL (R&D Systems) and 1 × 10^3^ RAW 264.7 cells and cultured for 7 days. The cells were then fixed in formalin and stained for TRAP using a TRAP staining kit (MilliporeSigma). TRAP^+^ cells containing 3 or more nuclei were counted as osteoclasts.

Human CD14^+^ monocytes sorted from the bone marrow of patients with MM were differentiated into osteoclasts with M-CSF (R&D Systems) and RANKL for 7 days, and CM were added to human CD14^+^ monocyte culture media (50%) for another 7 days. The cells were then fixed in formalin and stained for TRAP using a TRAP staining kit. TRAP^+^ cells containing 3 or more nuclei were counted as osteoclasts.

### Treatment of BCMA–CST6–CAR-T cells using MM mouse models.

Ten-week-old female NOD.*Cg-Prkdc^scid^Il2rg^tm1Wjl^*/SzJ (NSG) mice (The Jackson Laboratory) were administered 1.5 × 10^6^ MM1.S cells, OPM2 cells, or H929 MM cells expressing luciferase via i.v. injection and randomly assigned to 3 groups (*n* = 5/group). On day 7 after MM cell injection, 1.5 × 10^6^ CAR-T cells were administered. d-luciferin was i.p. injected (150 mg/kg), and bioluminescence images were acquired 10 minutes later using the IVIS Spectrum In Vivo Imaging system (PerkinElmer). Myeloma progression was monitored every 7 days, and on day 35 after tumor cell inoculation, when mice started to develop paraplegia, the mice were sacrificed. Mouse tibias were collected for μCT and TRAP staining of osteoclasts.

We repeated the in vivo experiment for observation of the disease progression curve. Myeloma progression was monitored by bioluminescence images every 7 days until the mice developed hind limb paralysis or the bioluminescence signal was more than 2 × 10^10^.

### Serum calcium and PTHrP analysis.

As a part of the euthanization, mice were terminally bled. The serum was separated and frozen at –80°C until calcium and PTHrP measurement. Serum calcium levels were measured using a calcium assay kit (colorimetric) (Abcam, ab102505). Serum PTHrP levels were measured using a Human PTHrP ELISA kit (MyBioSource, MBS451344).

### μCT.

Tibias from NSG mice were fixed in 10% neutral-buffered formalin for 2 days. A SkyScan 1272 scanner (Bruker) was used for μCT of each mouse tibia. The scans were acquired using the following imaging parameters: voltage, 60 kV; current, 166 uA; filter, aluminum (Al) with a thickness of 0.5 mm; and pixel size, 10 μm. After the tibia scans were acquired, the images were reconstructed using the Skyscan NRecon program with a beam hardening correction of 40. The microarchitecture of trabecular bone was examined using Skyscan CT Analyzer software.

### Bone histomorphometry.

After the μCT scans, the same tibias were decalcified in a 5% EDTA solution (pH 7.0) at room temperature for 7 days. Subsequently, they were embedded in paraffin. Bone sections, each 5 μm thick, were then prepared and stained using H&E and TRAP, utilizing the Leukocyte Acid Phosphatase Kit from MilliporeSigma. Histomorphometric analyses used OsteoMeasure software (OsteoMetrics) with a Zeiss Axioskop2 microscope from Carl Zeiss.

### Statistics.

All data were analyzed with GraphPad Prism 9 (GraphPad Software) and are presented as the mean ± SD unless otherwise indicated. For statistical analyses, a 2-tailed Student’s *t* test was performed when only 2 groups were compared. One-way ANOVA was used to determine the statistically significant difference for multiple-group comparisons. The log-rank test was used to determine statistically significant differences in the in vivo disease progression experiments. A *P* value of 0.05 or less was considered significant.

### Study approval.

All animal procedures adhered to a protocol (no. 3997) approved by the local IACUC at UAMS. Deidentified PBMCs and BMMCs were obtained from the UAMS Myeloma Center Tissue Biorepository and Procurement Core. Signed IRB-approved informed consent forms are kept on record in the Myeloma Center Tissue Biorepository and Procurement Core. The UAMS IRB approved these research studies (protocol nos. 261817 and 261821).

### Data availability.

The authors are committed to the open sharing of data. Underlying data for the figures are available in the Supplemental Supporting Data Values file and from the corresponding author upon request.

## Author contributions

FS and YC performed the experiments, collected and analyzed data, generated the figures, and wrote and edited the manuscript. JRC, VW, DEM, HX, DG, SAH, CS, ST, MZ, FVR, GT, and JDS reviewed the data, analyzed and interpreted data, and revised the manuscript. FZ designed and supervised this study, collected and analyzed data, and wrote and edited the manuscript. All authors discussed the results and commented on the manuscript.

## Supplementary Material

Supplemental data

Supporting data values

## Version 1 10/26/2023

In-Press Preview

## Version 2 01/02/2024

Electronic publication
