# Supplementary material for: BCMA- and CST6-specific CAR T cells lyse multiple myeloma cells and suppress murine osteolytic lesions
Source: J Clin Invest. 2024 Jan 2;134(1):e171396. doi: 10.1172/JCI171396 (PMC10760955; doi:10.1172/JCI171396)
Supplement: Supplemental data [file jci-134-171396-s077.pdf]

Supplemental material for “BCMA- and CST6-specific CAR T cells lyse multiple myeloma cells and suppress murine osteolytic lesions”

Table of Contents

Supplement	Title	page
Supplemental Figure1	Characteristics of BCMA-CST6-CAR-T cells	2
Supplemental Figure2	BCMA-CST6-CAR-T cells suppress formation of osteoclasts in vitro.	3-4
Supplemental Figure3	BCMA-CST6-CAR-T cells decrease osteolytic lesions in MM-burden mice.	5-6

Supplemental Figure 1

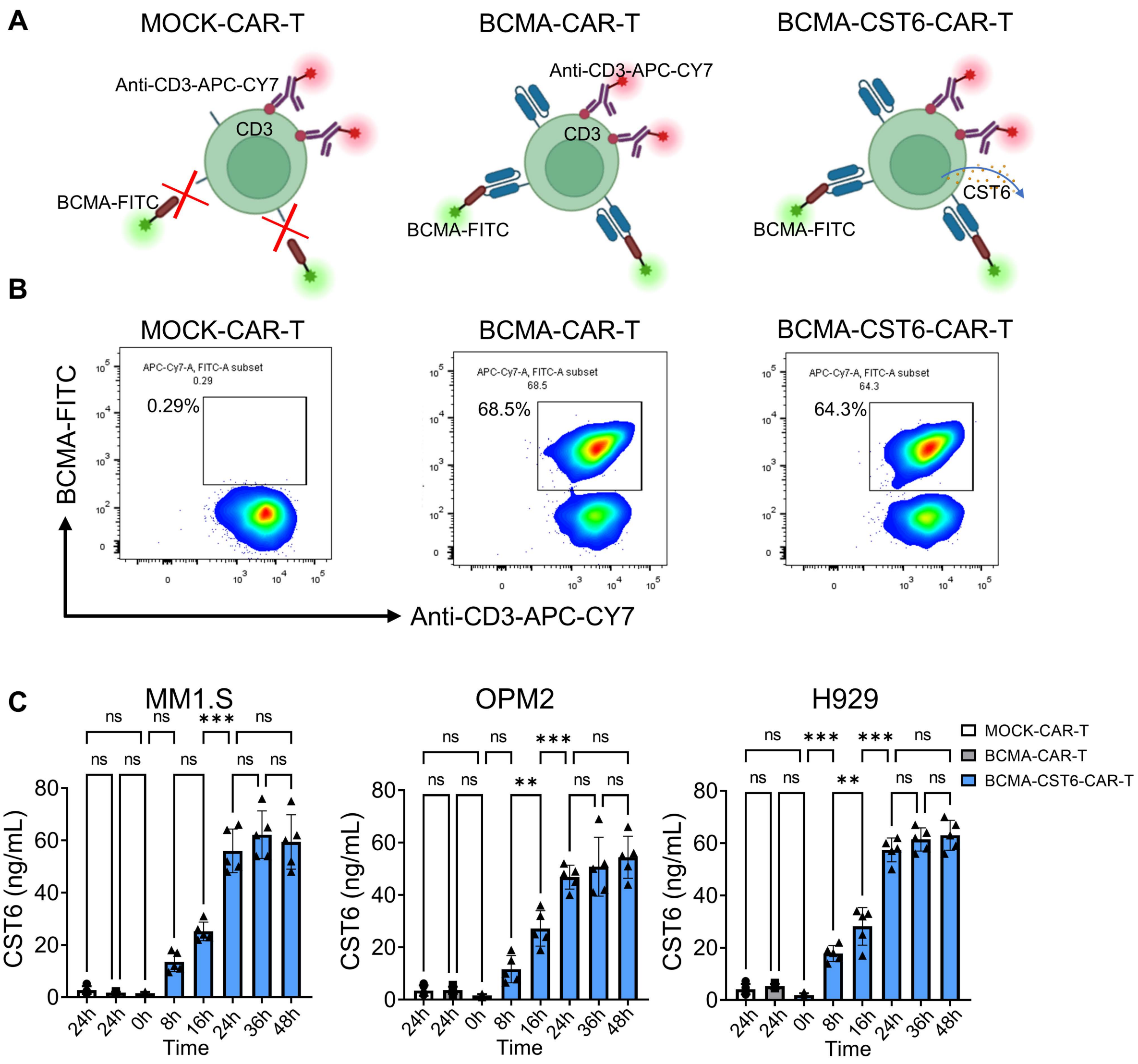

Supplemental Figure 1: Characteristics of BCMA-CST6-CAR-T cells. (A) Schematic of the flow cytometry assay designed to test CAR-T cell-specific binding. **(B)** FITC-Labeled Human BCMA was used to evaluate the binding activity of CAR-T cells with human BCMA. Flow cytometry was used to detect the fluorescent light signals. **(C)** CST6 concentrations on supernatants were detected at the E/T ratio was 5:1 at different time points (0h, 8h, 16h, 24h, 36h, 48h) of co-culture (*n* = 5). Data represented mean ± SD. One-way ANOVA was used for statistical analysis. ∗∗∗*P* < 0.001, ∗∗*P* < 0.01, ns = *P* > 0.05.

Supplemental Figure 2

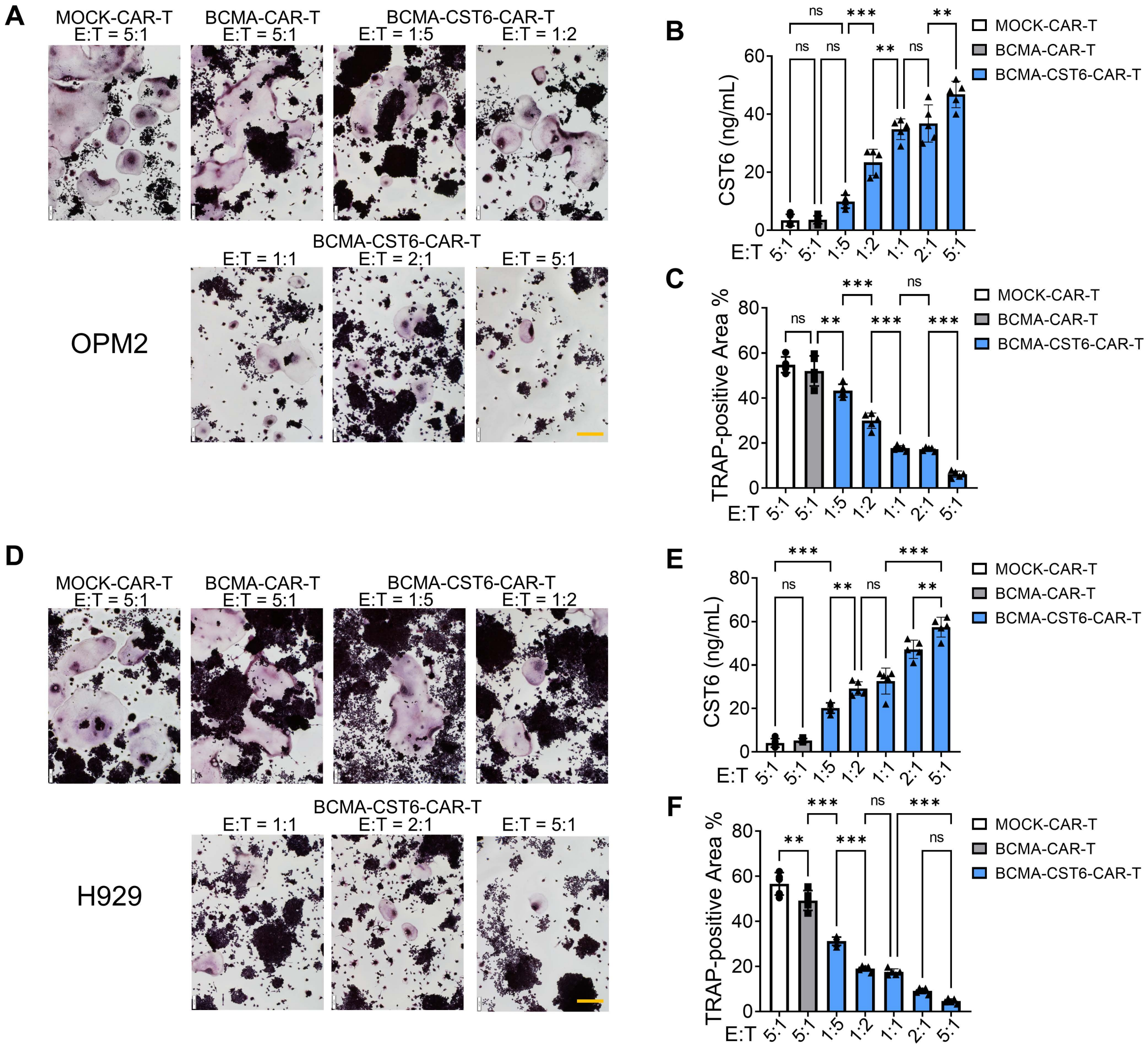

Supplemental Figure 2: BCMA-CST6-CAR-T cells suppress formation of osteoclasts in vitro. (A) CAR-T cells were incubated with OPM2 cells at ratios of 1:5 to 5:1 for 24 h and conditioned media were collected and added into RAW 264.7 cells with RANKL. On day 7, osteoclasts were stained with TRAP solution (*n* = 5, representative result from 5 independent experiments). Scale Bar = 200 μm. **(B)** CST6 concentrations on supernatants were detected at the E/T ratios from 1:5 to 5:1 after 24 hours of co-culture with OPM2 (*n* = 5). **(C)** Bar-plots presented quantifications of TRAP-positive area of (A) (*n* = 5). **(D)** CAR-T cells were incubated with H929 cells at the E/T ratios of 1:5 to 5:1 for 24 h and conditioned media were collected and added into RAW 264.7 cells with RANKL. On day 7, osteoclasts were stained with TRAP solution (*n* = 5, representative result from 5 independent experiments). Scale Bar = 200 μm. **(E)**

CST6 concentrations on supernatants were detected at the E/T ratios from 1:5 to 5:1 after 24 hours of co-culture with H929 (n = 5). **(F)** Bar-plots presented quantifications of TRAP-positive area of (D) (n = 5). Data represented mean ± SD. $***P < 0.001$, $**P < 0.01$, ns = $P > 0.05$.

Supplemental Figure 3

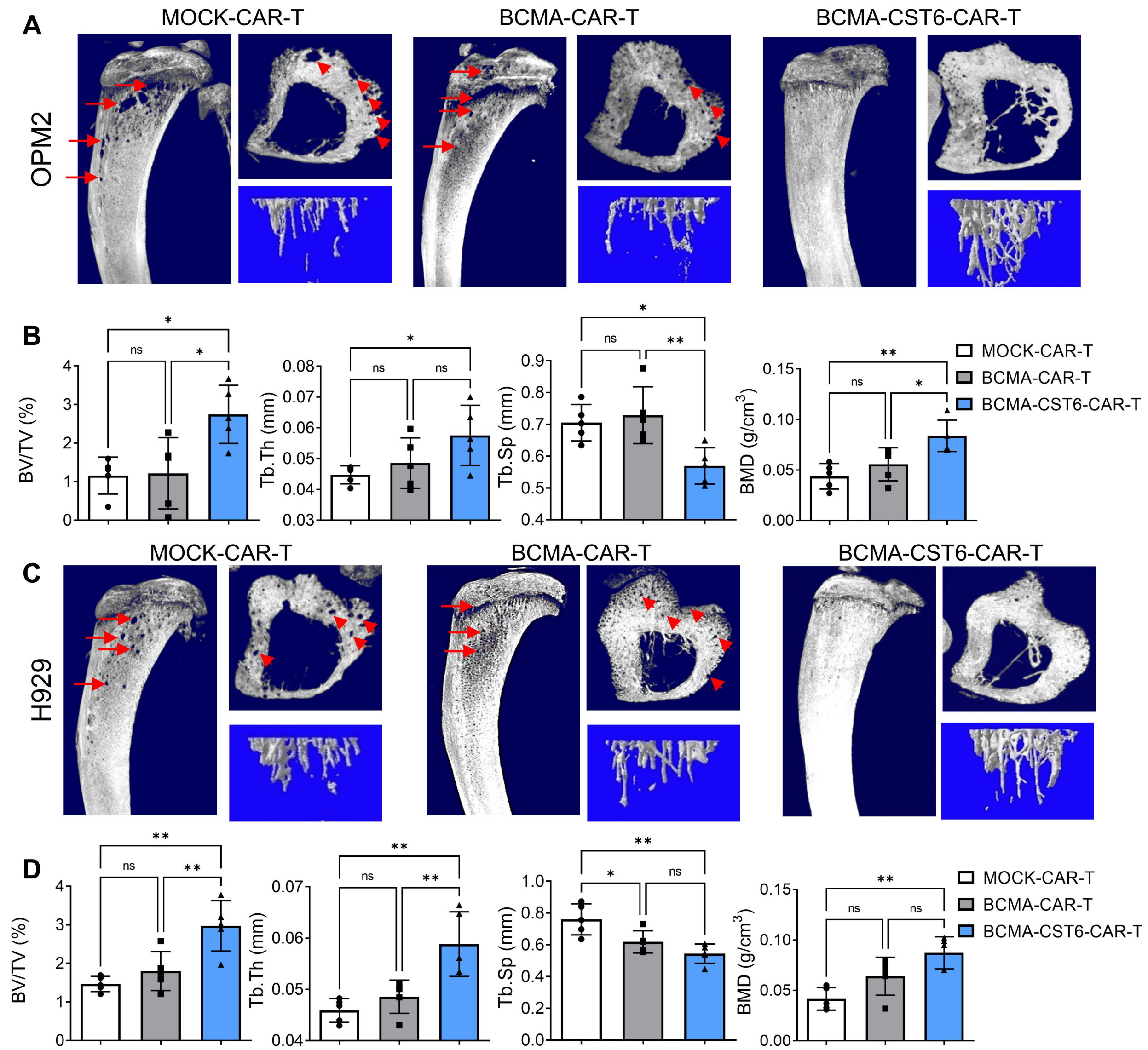

Supplemental Figure 3: BCMA-CST6-CAR-T cells decrease osteolytic lesions in MM-burden mice. (A) Reconstructed μCT images of tibia sagittal sections showed bone lytic lesions (indicated with arrows) and trabecular architecture of OPM2 xenograft model (*n* = 5, representative result from 5 mice). **(B)** Bar plots presented number of bone lytic lesions on the right medial tibia surface, trabecular bone parameters, trabecular bone volume over total volume (BV/TV); trabecular thickness (Tb.Th); bone mineral density (BMD); trabecular separation (Tb.Sp) of OPM2 xenograft model (*n* = 5). **(C)** Reconstructed μCT images of tibia sagittal sections showed bone lytic lesions (indicated with arrows) and trabecular architecture of H929 xenograft model (*n* = 5, representative result from 5 mice). **(D)** Bar plots presented the

number of bone lytic lesions on the right medial tibia surface, trabecular bone parameters, trabecular BV/TV; Tb.Th; BMD; Tb.Sp of H929 xenograft model (n = 5). Data represented mean ± SD. $*P < 0.05$, $**P < 0.01$, ns = $P > 0.05$.
